# Timing and Composition of Last Meal before Bedtime Affect Sleep Parameters of Night Workers

**DOI:** 10.3390/clockssleep3040038

**Published:** 2021-10-14

**Authors:** Luciana F. R. Nogueira, Pollyanna Pellegrino, José Cipolla-Neto, Claudia R. C. Moreno, Elaine C. Marqueze

**Affiliations:** 1 Department of Epidemiology, Public Health Graduate Program, Catholic University of Santos, Santos 11015-002, SP, Brazil; nogueira.lfr@gmail.com (L.F.R.N.); pollyanna.pellegrino@gmail.com (P.P.); 2 Department of Physiology and Biophysics, School of Public Health, University of São Paulo, São Paulo 01246-904, SP, Brazil; cipolla@icb.usp.br; 3 College of Health Sciences, Abu Dhabi University, Zayed City 999041, United Arab Emirates; 4 Department of Health, Life Cycles and Society, School of Public Health, University of São Paulo, São Paulo 01246-904, SP, Brazil; crmoreno@usp.br; 5 Stress Research Institute, Department of Psychology, Stockholm University, SE-106 91 Stockholm, Sweden

**Keywords:** feeding behavior, nutrients, sleep, night-shift work, nursing personnel

## Abstract

Night workers tend to eat irregularly, both in terms of meal times and composition. The disruption in energy metabolism caused by inappropriate eating habits can negatively affect the sleep quality of these individuals. The objectives of this study were to determine the interval between the last meal and bedtime and its relationship with both diurnal and nocturnal sleep parameters, as well as to evaluate the association of the adequacy of this meal with sleep parameters. The analyses were carried out for a usual sleep routine on a workday and a day off. This cross-sectional study was part of a controlled, randomized, double-blind, crossover clinical trial. The sample comprised 30 female nursing professionals who worked permanent night shifts of 12 × 36 h. Timing and composition of the last meal were obtained from food diaries, and sleep parameters were collected via actigraphy. On multiple linear regression analysis, every hour decrease in the interval between the last meal and sleep onset there was an increase of 0.39 h on diurnal sleep duration. Regarding food intake, every 1 g of fat and 1 g of carbohydrate consumed was associated with an increase in diurnal sleep onset latency of 0.13 h and 0.02 h, respectively. These findings suggest that both timing and composition of the last meal before bedtime may be potential key factors for good diurnal and nocturnal sleep among night-shift workers.

## 1. Introduction

Heterogeneous findings regarding the influence of diet composition on the sleep of night workers may be associated with meal timing [1]. This issue is attracting growing research interest and is especially relevant in the context of dietary patterns of permanent night workers, a group subject to chronic circadian misalignment [2].

Most studies on the subject have failed to compare differences in patterns of daily calorie intake between night workers and permanent day workers. These groups differ in the distribution of meals over a 24-h period, where night workers tend to eat irregularly during their shifts, consuming carbohydrates and high-fat snacks [3,4]. However, the metabolism of carbohydrates and lipids is especially impaired during the night [5], where the intake of these nutrients at biologically inappropriate times has deleterious effects on the proper functioning of the metabolism and quality of sleep [6].

The processes of digestion and absorption of nutrients are regulated by hormones that express circadian rhythms, such as insulin, leptin, and ghrelin. Once secreted, these hormones act as metabolic signals for peripheral tissues and influence peripheral oscillators [4]. Since humans are diurnal animals, food intake at night adversely affects the homeostasis of energy metabolism [5,7]. As these oscillators are closely linked to the sleep-wake cycle, the desynchronization of the feeding-fasting cycle for central and peripheral oscillators can negatively affect sleep by disrupting energy metabolism [8].

Previous findings show that night workers tend to display poorer adherence to nutritional recommendations [9]. However, new evidence has suggested that controlling the timing of food intake represents a potential therapeutic approach in the context of preventing overweight and metabolic disorders [10,11]. Current trends indicate that this relationship, which is potentially mediated by sleep duration since childhood [12], is explained by the hypothesis that both sleep and metabolism share, and are modulated by, the same hypothalamic circuits [13].

A review on nutritional deficits of nursing professionals revealed that the food intake of night workers is governed more by working hours, i.e., opportunities to eat during the shift than by hunger [14]. Torre et al. [11], in a recent evaluation of the eating habits of night workers, found that both the composition and timing of meals varied according to working hours, where the recovery day (after a night shift) proved the most affected. Also, a previous study has shown that meal content had a BMI-mediated effect on sleep, with obese workers the most affected [15].

In addition to nutrients, mealtimes can also affect sleep through circadian changes responsible for hormonal regulation of the central and metabolic nervous system [16]. When food consumption occurs close to bedtime, it leads to a shorter sleep duration and to a change in the time of sleep stages [17]. However, the time interval during which food interferes with sleep, and the nature of this interference, have yet to be elucidated [18]. Given the above, our hypothesis holds that the shorter the time interval between last meal and bedtime, and the more inadequate the content of this meal, the worse the sleep outcomes. Therefore, the objectives of this study were to determine the interval between last meal and bedtime, and its relationship to both diurnal and nocturnal sleep parameters, as well as to evaluate the association of the adequacy of this meal with sleep characteristics in overweight night shift workers.

## 2. Results

The diurnal sleep sample comprised a total of 30 participants and the nocturnal sleep sample comprised 22 participants (see Methods). The mean age of participants was 39.2 years (SE 1.0 years). Overall, participants were predominantly nurses (52.6%), married (63.2%), and had worked the night shift for more than five years (52.6%). Most participants were educated to postgraduate level (42.1%). The main reasons for choosing this work regimen were to reconcile work with home and childcare (44.7%) and to supplement income (21.1%). Most participants were non-smokers (89.5%) and consumed alcohol only on special occasions (65.8%). Mean body mass index (BMI) was 30 kg/m^2^ (SE 0.5 kg/m^2^).

**Figure 1:**
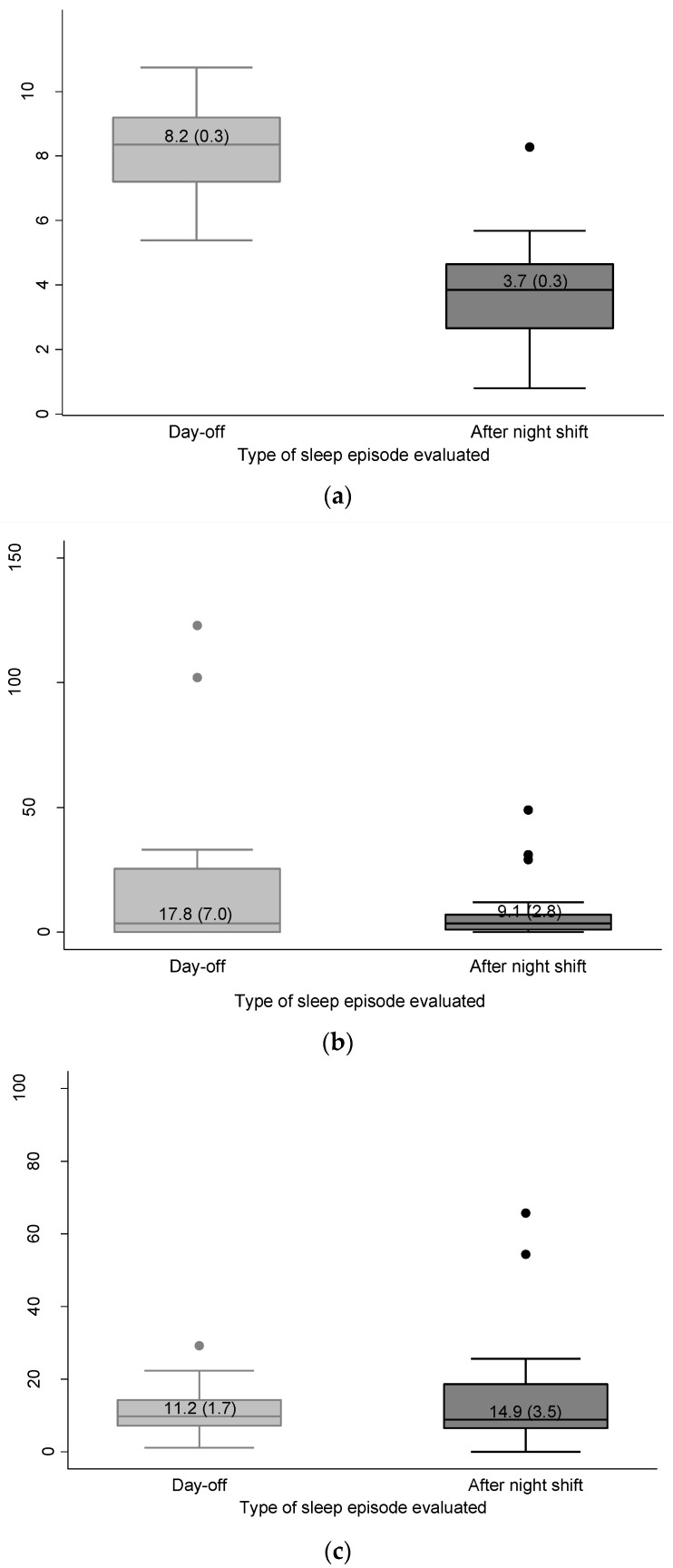
Sleep parameters obtained by actigraphy after night work (diurnal sleep, *n* = 22) and on day-off (nocturnal sleep, *n* = 22): (**a**) sleep duration (hours) (Paired *t*-test, *p* < 0.01) (**b**) sleep onset latency (minutes) (Wilcoxon *p* = 0.87) and (**c**) wake-up after sleep onset (%) (Wilcoxon *p* = 1.00). White lines represent means, gray boxes represent standard errors, and whiskers represent 95% confidence intervals.

Mean sleep duration was higher on the day-off (nocturnal sleep) than after the night shift (diurnal sleep). There was no significant difference for mean sleep onset latency (SOL) and mean wake-up after sleep onset (WASO) between the two groups (after night work and day-off) (Figure 1).

**Figure 2:**
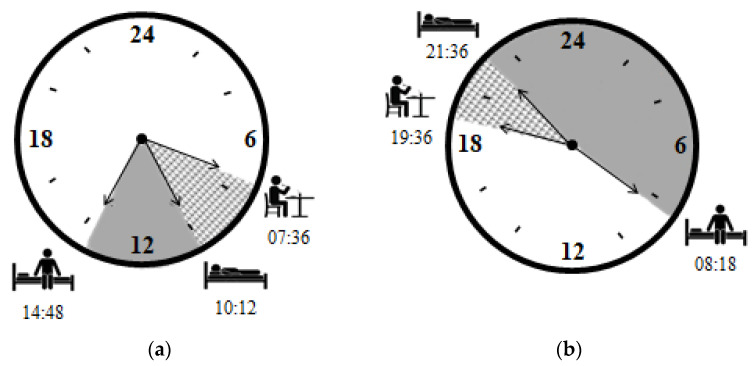
Mean times (clock time) of last meal before bedtime, of sleep onset and of getting up after: (**a**) night shift (*n* = 22); and (**b**) day-off (*n* = 22).

The last meal after night work occurred 2.6 h (SE 0.3 h) before the participants initiated sleep. On the day-off, this interval was 2 h (SE 0.3 h). There were no significant differences between the intervals for the days evaluated (Paired *t*-test *p* = 0.52) (Figure 2).

Results for mean daily intake of macronutrients on workdays and days-off reveal that both were in line with nutritional recommendations: workdays (fat: 31.1%, SE 1.3%; carbohydrate: 49.8%, SE 1.7%; protein: 18.3%, SE 1%); days-off (fat: 29.4%, SE 1.3%; carbohydrate: 51.5%, SE 1.8%, protein: 18%, SE 1.1%). Analysis of the composition of last meal before bedtime showed that mean percentages of macronutrients were adequate: workdays (fat: 26.4%, SE 2.5%; carbohydrate: 60.5%, SE 3.2%; protein: 14.9%, SE 1.8%); days-off (fat: 28.5%, SE 2.7%; carbohydrate: 58.5%, SE 3.7%; protein: 14.7%, SE 2.1%).

**Figure 3:**
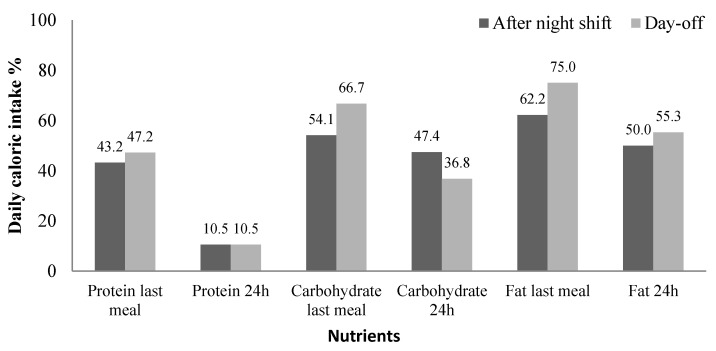
Percentage of participants with inadequate daily macronutrient intake for 24 h period and last meal before bed, after night shift (*n* = 30), and on day-off (*n* = 22). *t*-test, *p* > 0.05.

There was no statistically significant difference between workday and the day-off for the 24-h period and last meal before bedtime. Although these results suggest macronutrient intake of participants was adequate, most participants had inadequate fat and carbohydrate consumption, both for the 24 h recording period, and last meal before bedtime after the night shift and on the day-off (Figure 3).

There was also no difference in total caloric intake after a night shift in comparison to day-off (1801 kcal vs. 1645.8 kcal, *t*-test *p* = 0.38). However, the proportion of calories provided by the last meal before the main episode of sleep was significantly higher on the day-off than after the night shift (25.5% vs. 15.2%, *t*-test *p* = 0.01).

### 2.1. Interval between Last Meal and Bedtime

On the multiple linear regression analysis of sleep duration on the workday, every hour decrease in interval between last meal and sleep onset was associated with an increase of 0.39 h on sleep duration (Table 1). No statistically significant results were found for the day-off.

On the multiple linear regression model of SOL on the day-off, no statistically significant results were found on the day-off. Nevertheless, on the crude linear regression for every one-hour increase in the interval between the last meal and sleep onset, there was an increase of 11.51 min in SOL (Table 2).

### 2.2. Composition of Last Meal

On the multiple linear regression analysis of SOL on the workday, for every 1 g of fat and 1 g of carbohydrate consumed there was an increase of 0.13 min and 0.02 min (borderline significance) on SOL, respectively (Table 3).

No statistically significant results for WASO were found on the workday or day-off.

## 3. Discussion

This study found that a shorter interval between the last meal and sleep onset was associated with an increase in the duration of diurnal sleep of overweight night shift workers. Additionally, higher macronutrient (fat and carbohydrate) consumption during the last meal before sleep onset was associated with higher sleep onset latency (SOL) for diurnal sleep. Lastly, a longer interval between the last meal and sleep onset was associated with increased SOL for nocturnal sleep in the crude model.

In a discussion paper by Lowden et al. [9], the authors observed that both meal content and timing are important for the nutritional management of night shift workers. More recently, previous studies have shown that energy metabolism exhibits circadian rhythms and that nutrient intake during the night may disrupt these rhythms [19]. In parallel, it is well established that sleep quality is associated with several hormonal and metabolic changes, including macronutrient modulation [20]. Therefore, it is hypothesized that eating at night affects sleep more than eating the same foods during the day. In this context, the current recommendation is that night shift workers should limit food intake during the night [21]. However, there is little information available in the literature on the impact of this recommendation on daytime sleep after night shifts.

Diurnal sleep is one of the factors promoting misalignment of internal biological clocks with the external environment; when associated with insufficient sleep, this may lead to temporal changes in the rhythm of hormonal and enzymatic activities responsible for metabolic regulation [22]. Moreover, the timing of food intake is a strong zeitgeber for peripheral circadian clocks, as is dietary composition [23]. Thus, these factors give rise to a scenario in which the organism is less able to cope with food, that is, to digest and use it as an energetic substrate [24]. Taking these factors together, the relationship between sleep duration and diet composition may be bidirectional, since sleep debt is associated with a higher intake of both fat and carbohydrate and vice-versa [25,26].

In this regard, the association found in the present study refers to daytime sleep after night work, which proved very short in comparison to the 7–9 h recommended for the adult population [27]. It is known that the sleep of night workers during the day is less restful and shorter compared to night sleep [22]. Therefore, the short duration of diurnal sleep in the present study deserves attention, since sleeping less than needed is associated with cardiometabolic diseases [28] and the diet contributes to the development of the condition [29].

A possible mechanism to explain this dietary influence is that the distribution of macronutrients can affect sleep continuously, promoting changes in neuroendocrine signals related to energy metabolism [28,30]. However, these differences in sleep patterns caused by the last meal highlight, as observed in a systematic review by Souza et al. [31], that night work has a greater influence on the timing of consumption than on total daily intake. Supporting this theory, a recent meta-analysis concluded that night work does not alter energy intake [32].

According to Kogevinas et al. [33], eating 2 h or less before initiating sleep contributes to negative health outcomes. Corroborating these findings, the present study found negative effects on sleep due to inadequate intake of macronutrients less than 2 h before bedtime. Unlike the present investigation, the analysis performed by Kogevinas et al. [33] evaluated only episodes of nocturnal sleep and was not performed exclusively with night workers. In fact, to our knowledge, the present study is the first to evaluate the effects of the last meal, both before diurnal and nocturnal sleep, on night shift workers alone.

The shorter sleep duration when the meal is taken a long time after the diurnal sleep episode might be related to feeling hungry. As Lowden et al. suggested, eating breakfast before going to sleep in the morning is a strategy to prevent awakening due to hunger [9]. Regarding meal content, Jansen et al. [29] found an inverted U-type curve when assessing the association between sleep duration and diet adequacies in adults. Therefore, as demonstrated by the results of the present study, inadequate consumption of nutrients, either below or above recommended values, seems to negatively impact sleep. This result reported by Jansen et al. [29] is consistent with the findings of the present study since the highest percentage of inadequacies in macronutrient intake was observed at the last meal.

The present study has some limitations. Although the participants were instructed to record food intake on typical days, we cannot guarantee that the pattern of distribution of macronutrients observed truly reflects their usual eating behaviors in everyday life. In addition, the adequacy of macronutrient intake at the last meal was calculated based on a recommendation originally intended to assess food intake over a 24-h period. However, it is important to highlight that the decision to adopt this recommendation was due to the absence of nutritional recommendations that take into account the timing of food intake, reiterating the need to establish consensus on this topic [34]. In addition, the physical and cognitive demands of the night work performed before the diurnal sleep episode, but not before nocturnal sleep (after a day-off) may have been a key factor determining sleep quality and should therefore be controlled in future studies. The difference in the diurnal and nocturnal sleep samples, which occurred because the day-off was not included in 10 days of data collected by actigraphy for 6 participants, may also represent a statistical bias for the results obtained. However, assessing sleep and dietary habits of night shift workers in real-life conditions, and evaluating the associations of meal timing and composition with both diurnal and nocturnal sleep, remain the main strengths of the study. Finally, the present study evaluated permanent night workers only, and hence results cannot be generalized to other types of shift workers [11,35,36].

In conclusion, the findings of this study suggest that both timing and the composition of the last meal before bedtime may be potential key factors for good diurnal and nocturnal sleep in night shift workers, and emphasize the need for nutritional guidelines for night shift workers.

## 4. Materials and Methods

### 4.1. Study Sample and Design

This cross-sectional study was part of a controlled, randomized, double-blind, crossover clinical trial in a large hospital situated in the city of São Paulo, São Paulo state, Brazil. Detailed information about the study design is provided elsewhere [37]. For the present study, involving nursing professionals (nurses and nursing technicians) who worked permanent night shifts of 12 × 36 h (12 h of night work, from 19:00 to 07:00 h, followed by 36 h of rest, with a day-off every 15 days), only the baseline data were analyzed. A total of 238 professionals were initially invited to participate in the study. Of this group, 152 did not meet the inclusion criteria and a further 40 refused to take part. Of the 46 subjects who met the criteria and agreed to participate in the study, 30 completed the actigraphy monitoring and food diaries at the study baseline.

To calculate the sample size a priori, the comparison test of three means and a significance level of 5% were used as a reference, with a sampling power of 80% for a sample size of 33 individuals. The final sample of the present study had a sampling power of 73% (G*Power^®^).

The inclusion criteria were: female gender; age 20–50 years; body mass index (BMI) ≥25 and <40 kg/m^2^; working the current night shift for at least six months; agreeing not to follow diets restricted in calories or food groups and/or change eating habits, as well as not to engage in new physical activities while participating in the study.

The exclusion criteria were: pregnant or lactating women; having infants under one year of age; eating disorders diagnosed by a physician; climacteric or menopause; holding a second nighttime job; regular use of medications or dietary supplements that influence sleep, alertness, or the circadian timing system; abusive use of drugs and alcohol; past and/or current history of neurological, psychiatric, circadian or sleep disorders; metabolic problems (except treated type 2 diabetes mellitus and dyslipidemia); cardiovascular diseases (except treated systemic arterial hypertension), inflammation and/or chronic infections diagnosed by a physician; anemia or >400 mL of blood donated in the three months preceding the study; major surgery in the six months preceding the study.

### 4.2. Data Collection and Processing

Baseline data collection was carried out from April to November 2018. Sociodemographic characteristics, as well as aspects related to work and health, were obtained using a self-administered questionnaire. The dependent variables were objective sleep data (mean values) obtained via actigraphy (ActTrust, Condor Instruments^®^) over 10 consecutive days [38]. The night shift and the day-off were not included in the original 10 days of data collection from actigraphy for two and eight participants, respectively, then the study was conducted with a diurnal sleep sample of 28 participants and a nocturnal sleep sample of 22 participants. For the present study, it was decided to use the sleep parameters obtained by actigraphy only on the days that food diaries (described above) were filled out. This approach ensured the sleep episode analyzed reflected the prior food consumption on the workday and day-off.

Concomitantly, participants were instructed to fill in sleep logs to complement the information and assist interpretation of the data obtained by actigraphy. The data were separated into diurnal sleep (after a night of work) and nocturnal sleep (the night following the day-off which occurred every 15 days). The parameters evaluated were sleep duration (hours), sleep onset latency (SOL) (minutes), and wake-up after sleep onset (WASO) (minutes).

The independent variables were timing and percentage distribution of macronutrients from the last meal before initiating sleep. Therefore, the participants were instructed to fill out food diaries on a workday and a day-off they considered typical. In both cases, the period for recording the food data was from 19:00 to 19:00 the next day. Participants were previously instructed to provide as much information as possible on the food and drinks consumed, including brands, ingredients used in homemade preparations, and meal timing. Portion sizes were estimated using household measures and subsequently converted to mass units of measurement (g) and capacity (mL), according to Pinheiro et al. [39]. The diaries were reviewed by a nutritionist to obtain additional clarifications, where necessary, and analyzed using the Nutrition Data System for Research software (NDSR, 2007).

Due to cultural differences between Brazilian and North American eating habits, the composition of typical preparations was manually added to the software database using the Brazilian Food Composition Table [40] and labels of industrialized products. To determine the interval between last meal and sleep onset, data on sleep onset was extracted from actigraphy. After night work, the last meal taken before diurnal sleep was considered for analysis, whereas on the day-off, the last meal before nocturnal sleep was considered.

The composition of the last meal was evaluated using the current Recommended Dietary Allowances (RDA) established by the National Academy of Sciences [41] and adopted by the Brazilian Society of Food and Nutrition [42]. The intake of macronutrients in the last meal before the main sleep episode was then classified as adequate or inadequate. The percentages of calories from each macronutrient classified as suitable were 25–35% for fats, 45–65% for carbohydrates, and 10–35% for proteins. Intakes were deemed inadequate when below the minimum limit or above the maximum limit of the aforementioned ranges. The decision to include both lower and higher-than-recommended intakes (i.e., 30 and 70% of carbohydrates) in the inadequate group was due to previous evidence on the association between sleep and diet in a representative sample of the North American adult population. Jansen et al. [29] observed that both lower and higher-than-recommended macronutrient intakes can negatively impact sleep outcomes. This decision was also based on a previous study on the same population analyzed in the present investigation [39]. The proportion of calories and macronutrients provided by the last meal in relation to daily intake was also calculated. Food diaries were filled in on a typical day of work and day-off, within the period of actigraphy monitoring.

### 4.3. Statistical Analysis

Data are expressed as mean and ± standard error (SE). Shapiro–Wilk’s test was performed to assess data distribution. Paired t-test was performed to compare sleep duration, as well as total caloric intake, percentage, and timing, for the last meal after the night shift (participants left work at 07:00 h) versus last meal on the day-off (sleep was nocturnal, therefore last evening meal was considered). To compare sleep onset latency (SOL) and the mean of wake-up after sleep onset (WASO) we performed the Wilcoxon test.

Analysis was performed using univariate and multiple linear regressions (general linear model), on which sleep duration, SOL, and WASO (on a workday and day-off), were dependent variables and β coefficients estimated for a 95% confidence interval. The models were constructed adopting timing and composition of the last meal (protein, fat, and carbohydrate) as independent variables, all adjusted for age, chronotype, and time working nights. For sleep duration, we performed a linear regression (general linear model) with normal distribution, and for SOL and WASO we performed a linear regression (general linear model) with Gamma distribution. Independent variables with *p* < 0.20 on the hypotheses tests were input to the multiple models, in decreasing order of statistical significance (backward stepwise technique). Statistical analyses were performed using Statistica 7 (TIBCO Software Inc., Palo Alto, CA, USA).

### 4.4. Ethical Aspects

Ethical issues related to research involving humans have been duly respected and informed consent was drafted in accordance with Resolution 466/2012. The study was approved by the Ethics Committees of the School of Public Health of the University of São Paulo (permit no 2.450.682, 20 December 2017) and the participating hospital (permit no 2.489.636, 7 February 2018). The protocol of the clinical trial is registered on the World Health Organization’s International Clinical Trials Registry Platform (UTN no U1111-1238-7395) and the Brazilian Registry of Clinical Trials (ReBEC–RBR-6pncm9).

## Figures and Tables

**Table 1:** Sleep duration after night shift (diurnal sleep) (*n* = 28).

Variables	Crude β	95% CI	Multiple β Adj ^&^	95% CI
Fat	−0.01	−0.02; 0.01	0.00	−0.01; 0.02
Carbohydrate	0.00	−0.01; 0.00	0.00	−0.01; 0.00
Protein	−0.01	−0.03; 0.00	−0.01	−0.02; 0.01
Interval between last meal and sleep onset	−0.32	−0.59; −0.05	−0.39	−0.63; −0.16

**^&^** Adjusted for age, chronotype and time (years) working nights.

**Table 2:** Sleep onset latency (SOL) on day-off (nocturnal sleep) (*n* = 22).

Variables	Crude β	95% CI	Multiple β Adj ^&^	95% CI
Fat	−0.10	−0.36; 0.16	0.09	−0.06; 0.24
Carbohydrate	−0.03	−0.16; 0.11	0.01	−0.10; 0.12
Protein	−0.18	−0.47; 0.10	0.11	−0.03; 0.25
The interval between last meal and sleep onset	11.51	3.25; 19.76	3.05	−6.41; 12.50

**^&^** Adjusted for age, chronotype, and time working nights.

**Table 3:** Sleep onset latency after night shift (diurnal sleep) (*n* = 28).

Variables	Crude β	95% CI	Multiple β Adj ^&^	95% CI
Fat	0.08	−0.07; 0.24	0.13	0.06; 0.20
Carbohydrate	0.06	0.01; 0.11	0.02	0.00; 0.05
Protein	0.03	−0.10; 0.15	0.02	−0.08; 0.12
Interval between last meal and sleep onset	−1.32	−2.99; 0.35	−0.44	−1.65; 0.77

**^&^** Adjusted for age, chronotype and time working nights.

## Data Availability

The data presented in this study are available on request from the corresponding author. The data are not publicly available due to privacy concerns.
